# Adaptation versus plastic responses to temperature, light, and nitrate availability in cultured snow algal strains

**DOI:** 10.1093/femsec/fiad088

**Published:** 2023-08-08

**Authors:** Emily L M Broadwell, Rachel E Pickford, Rupert G Perkins, Fotis Sgouridis, Christopher J Williamson

**Affiliations:** School of Geographical Sciences, University of Bristol, University Road, Bristol, BS8 1SS, United Kingdom; School of Geographical Sciences, University of Bristol, University Road, Bristol, BS8 1SS, United Kingdom; School of Earth and Environmental Sciences, Cardiff University, Main Building, Park Place, Cardiff, CF10 3AT, United Kingdom; School of Geographical Sciences, University of Bristol, University Road, Bristol, BS8 1SS, United Kingdom; School of Geographical Sciences, University of Bristol, University Road, Bristol, BS8 1SS, United Kingdom

**Keywords:** photophysiology, psychrophile, snow algae, stoichiometry

## Abstract

Snow algal blooms are widespread, dominating low temperature, high light, and oligotrophic melting snowpacks. Here, we assessed the photophysiological and cellular stoichiometric responses of snow algal genera *Chloromonas* spp. and *Microglena* spp. in their vegetative life stage isolated from the Arctic and Antarctic to gradients in temperature (5 – 15°C), nitrate availability (1 – 10 µmol L^−1^), and light (50 and 500 µmol photons m^−2^ s^−1^). When grown under gradients in temperature, measured snow algal strains displayed Fv/Fm values increased by ∼115% and electron transport rates decreased by ∼50% at 5°C compared to 10 and 15°C, demonstrating how low temperatures can mimic high light impacts to photophysiology. When using carrying capacity as opposed to growth rate as a metric for determining the temperature optima, these snow algal strains can be defined as psychrophilic, with carrying capacities ∼90% higher at 5°C than warmer temperatures. All strains approached Redfield C:N stoichiometry when cultured under nutrient replete conditions regardless of temperature (5.7 ± 0.4 across all strains), whereas significant increases in C:N were apparent when strains were cultured under nitrate concentrations that reflected *in situ* conditions (17.8 ± 5.9). Intra-specific responses in photophysiology were apparent under high light with *Chloromonas* spp. more capable of acclimating to higher light intensities. These findings suggest that *in situ* conditions are not optimal for the studied snow algal strains, but they are able to dynamically adjust both their photochemistry and stoichiometry to acclimate to these conditions.

## Teaser

Laboratory incubations of isolated snow algal species have highlighted diverse plastic responses to gradients in temperature, light, and nitrate availability.

## Introduction

Snowpack and supraglacial ice surface environments are home to a diverse assemblage of microorganisms with predominately Chlorophyte snow algae and Streptophyte glacier algae acting as the prevalent photoautotrophs (Anesio and Laybourn-Parry 2012, Lutz et al. 2016, Anesio et al. 2017, Hoham and Remias 2020). These algal groups form widespread blooms across the cryosphere [i.e. North America (Hamilton and Havig 2017), Svalbard (Lutz et al. 2017), Europe (Procházková et al. 2019), and Antarctica (Soto et al. 2020)], when light and liquid water are available to drive photosynthesis during spring/summer ablation seasons (Hoham and Duval 2001, Stibal et al. 2012). Snow algal species are distributed across the Chlorophytes (Remias et al. 2010, Becker 2013) with key genera including *Chlamydomonas* spp., *Chloromonas* spp., and *Microglena* spp. (Hoham and Remias 2020, Soto et al. 2020). Most of these algae are considered freshwater psychrophilic snowpack specialists, having optimal growth at temperatures below 10°C (Lewis and McCourt 2004, Cvetkovska et al. 2017), however, some species have been reported as psychrotolerant, displaying the ability to grow under snowpack conditions, but showing higher growth rates at higher temperatures (Hoham 1975, Ling 2001, Hoham et al. 2008, Barcytė et al. 2018).

Snow algae bloom in an ephemeral snowpack environment, experiencing low temperatures, variable liquid water and nutrient availabilities, and high light intensities (Gorton et al. 2001, Techel and Pielmeier 2011, Rivas et al. 2016, Ren et al. 2019, Hoham and Remias 2020). Temperatures characteristic of ablating snowpacks are stable at or just above freezing during the ablation period, with the insulating properties of snow resulting in temperatures rarely shifting more than 1°C (Stibal et al. 2007, Burns et al. 2014, Maccario et al. 2015, Rivas et al. 2016). Such consistently low temperatures mean liquid water is not always available, varying from 0.5% to 15% of the snowpack (Techel and Pielmeier 2011), and thus microbial communities must be able to tolerate frequent desiccation. Nutrient availability of the snowpack is also variable across the ablation season, with 80% of the solutes present being released within the first 30% of meltwater (Kuhn 2001) during an initial ‘ionic pulse’ (Costa et al. 2017). Nutrient concentrations are generally oligotrophic across seasons and regions, with average nitrate and ammonia ∼5 µmol L^−1^ and 10 µmol L^−1^, respectively in Arctic (Larose et al. 2013) and Antarctic (Hodson 2006) snowpacks during the ablation season. Phosphate availability is also low, with snowpacks sampled in the Antarctic (Dubnick et al. 2017) and Rockies (Hamilton and Havig 2017) rarely containing levels above detection limits (0.24 µmol L^−1^ and 0.52 µmol L^−1^, respectively). On the snow surface, light intensities are dynamic, with photosynthetically active radiation (PAR) reaching 2000 – 3000µmol photons m^−2^ s^−1^ and UV 30% greater than sea level in alpine regions (Gorton et al. 2001, Morgan-Kiss et al. 2006). Within snowpacks, light attenuation with depth results in a notable drop in PAR, with intensity at 1 cm depth ~25% – 50% of that measured at the surface depending on prevalent weather conditions (Gorton et al. 2001, Stibal et al. 2007).

To-date, studies examining the impact of temperature on snow algae have focused on growth dynamics, with *Chloromonas* spp. trending towards faster growth rates but lower overall carrying capacities (*K*) with warmer temperatures (Hoham 1975, Hoham et al. 2008), demonstrating a balance between thermodynamic and biological processes within their cells. Research into responses to nutrient availability has focused primarily on metabolite production, with consideration to biotechnological applications. For example, nitrate and phosphate limitation drive upregulation of carbohydrate and fatty acid production in the snow alga *Chlamydomonas nivalis* to balance corresponding declines in amino and organic acid production under nutrient limitation (Lu et al. 2016). In other Chlorophyte snow algal strains, nitrate limitation has been shown to stimulate the production of nitrogen-free secondary carotenoid pigments, resulting in a change in bulk cellular colouration (Leya et al. 2009). *In situ* research into snow algal stoichiometry has found C:N ratios ranging from 16 to 33 (Spijkerman et al. 2012), much higher than the ‘optimal’ Redfield ratio of 6.6 (Redfield 1958).

As the quantity and quality of PAR available to snow algae is dynamic in both the short and long term, photosystems need to be flexible to utilize light efficiently, alongside preventing excess light from causing cellular damage (Remias et al. 2005, Procházková et al. 2023). When exposed to a continuous high light, chlorophytes have been shown to downsize the size of their antenna complex after several hours, such that cells still absorb adequate light while protecting PSII from photodamage, with the reverse true for long-term, low light acclimation (Melis 1991). Evidence of these photoacclimatory mechanisms have been recorded in a number of snow algal species through pulse amplitude modulated (PAM) fluorometry, with changes in both the efficiency of light use and the light intensity at which the PSII becomes saturated when the cells are exposed to varying light intensities (Procházková et al. 2019, Soto et al. 2020).

Despite many studies on snow algal responses to key abiotic stressors (i.e. Hoham et al. 2009, Leya et al. 2009), few have assessed responses to more realistic, multistressor conditions, nor potential inter- or intraspecific responses. Research into other cryospheric terrestrial microorganisms, including red snow algae (Segawa et al. 2018), has identified endemism (Vincent 2000, Convey 2010, Vyverman et al. 2010, Segawa et al. 2017), but it is not yet known if similar endemism exists across other snow algal species, nor the role of endemism in modulating responses to abiotic stressors. Here, we assessed the growth, cellular stoichiometric, and photophysiological capabilities of two snow algal genera (*Microglena* spp. and *Chloromonas* spp.) isolated from both the Arctic and Antarctic in their vegetative life stage. To identify temperature optima, growth, cellular stoichiometry, and photophysiology were monitored across 5, 10, and 15°C under nutrient replete conditions. Subsequently, multistressor responses to nitrate availability (1, 5, and 10 µmol L^−1^; Hodson 2006) and light intensity (50 and 500 µmol photons m^−2^ s^−1^) were assessed.

## Materials and methods

In total, two series of incubations were conducted to determine the growth, photophysiological, and stoichiometric responses of multiple snow algal strains to temperature gradients and subsequently variations in nitrate availability and light intensity. Incubations were performed with strains of snow algae from two genera, and on replicates of each strain isolated from both Arctic and Antarctic environments.

### Snow algal strains

A total of two genera of snow algae both isolated from King George Island, Antarctica and Spitsbergen, Svalbard were investigated during the present study (Table 1). Clonal algal cultures were acquired from the Culture Collection of Cryophilic Algae (CCCryo) at the Fraunhofer-IBMT in Potsdam (see Table 1 for strain numbers and origin). Cultures were transported to the Low Temperature Experimental Facility (LOWTEX) at the University of Bristol and stored in vented culture flasks (Corning, New York, USA) with nutrient replete 3N-BBM prior to experimentation (Bischoff and Bold 1963, Andersen 2005).

**Table 1.** Algal species, sample origin and location details, and associated CCCryo culture strain number. All cultures were isolated from snow fields in the given locations.

Species	Sample location	Strain number
*Microglena* cf. sp. -002b	East of Flåtjørna (195 m), Kongsfjorden, Ny-Ålesund, Spitsbergen, Svalbard.	038–99
*Microglena* cf. sp. -002b	North of Artigas Base freshwater lake, Fildes Peninsula, Maxwell Bay, King George Island, South Shetland Islands, Antarctica.	266–06
*Chloromonas* sp. (formerly *Chloromonas pichinchae*)	Mountain north of Kvalhovden, west of Storfjorden, southeast in Heer Land, Spitsbergen, Svalbard.	192–04
*Chloromonas* sp. (formerly *Chloromonas pichinchae*)	South-eastern coastline of Barton Peninsula towards Potter Cove, south of Fourcade glacier, King George Island, South Shetland Islands, Antarctica.	261–06

### Incubation design

#### Temperature incubations

Though snow algal species are generally regarded as psychrophilic, with optimal growth temperatures below 15°C, some are considered psychrotolerant, exhibiting preferential growth at higher temperatures (Hoham and Remias 2020). To assess the selected strains, an initial incubation experiment was conducted with all strains to monitor responses across a range of temperatures (5 – 15°C) under nutrient replete conditions (3N-BBM) and at a consistent light intensity of 50 µmol photons m^−2^ s^−1^. This data provided important context for our subsequent incubations where temperature was held constant around optimal growth conditions (4°C), while nitrate availability and light intensity were varied.

Temperature incubations were conducted at 5, 10, and 15°C using model 305a LMS incubators (LMS, Sevenoaks, UK) housed within the LOWTEX facility at the University of Bristol. This temperature range was selected to ascertain the temperature optima for these strains, determining whether they can be better defined as psychrophilic or psychrotolerant. To establish incubations, 5 mL of stock culture of each strain previously maintained under CCCryo recommended growth conditions (3N-BBM, 4°C, 50 µmol photons m^−2^ s^−1^) was inoculated into 45 mL 3N-BBM within a 50 mL Corning culture flask with a vented cap (Corning). A total of *N* = 8 replicates were established per algal strain and temperature treatment. Irradiance during incubations was provided by OSRAM L 8 W/535 fluorescent tubes (OSRAM, Munich, Germany) with a mean light intensity of 50 µmol photons m^−2^ s^−1^ provided on a 16:8 h L:D cycle. Throughout incubations, sampling for determination of algal abundance (cells mL^−1^) and biovolume (µm^3^ per cell) was conducted three times per week on 50 µL of each culture sampled using a sterile plastic tip on a manual displacement pipette after thorough homogenization. Following subsampling, sample flasks were repositioned randomly within incubators to ensure even light distribution. Cell abundance (see below) was used to identify exponential and stationary growth phases of each strain/temperature treatment, during which destructive sampling of *N* = 4 replicates was undertaken for photophysiological and stoichiometric analyses (see below).

#### Nutrient and light incubations

Following temperature incubations, the growth, photophysiological and stoichiometric responses of all algal strains were tested in response to gradients of nitrate availability and light, while temperature was held constant at 4°C. For these incubations, 4°C was chosen given that all strains demonstrated growth optima at lower temperatures during this study. Although snowpack conditions are often just above freezing (Burns et al. 2014), temperatures below 4°C were avoided here to prevent freezing of the cultures during incubations. For each algal species/strain, incubations were conducted in three 1000-OD-MIX algal multicultivator incubators (Photon Systems Instruments, Czech Republic), which allowed for specific wavelengths and light intensities to be applied and for the constant oxygenation of cultures. All incubators were maintained in temperature-controlled rooms within LOWTEX throughout the incubations. Each of the three multicultivators housed *N* = 8 replicate incubations of one nitrate treatment (Low, Medium, or High; Table 2), with all other nutrients maintained at replete concentrations as per standard BBM (Bischoff and Bold 1963, Andersen 2005). Nitrate treatments (ranging 1 – 10µmol L^−1^; Table 2) were selected to replicate the changes in nitrate availability apparent across the ablation season within melting snowpacks on top of supraglacial ice surfaces, shown to range 1 – 10 µmol L^−1^*in situ*, with higher concentrations experienced at the onset of melt (Hodson 2006, Telling et al. 2014, Holland et al. 2022). Nitrate assimilation has also been linked to light intensity in green algae (Aparicio and Quiñones 1991), as well as increased nitrate assimilation rates in snow algae compared to ammonia (Jones 1999). Though nitrate concentrations were not available for the specific sites where the cultures were isolated from, estimates from comparable snowpack environments formed the basis for our treatment conditions (Table 2).

**Table 2.** Nitrate concentrations used to reflect changing snowpack environments for the incubations as well as the nutrient replete 3N-BBM media used for the nutrient replete temperature incubations.

Media	Nitrate conc	Environment	References
Low NO_3_	1 µmol L^−1^	Supraglacial ice	Telling et al. (2014)
Medium NO_3_	5 µmol L^−1^	Fresh snow	Hodson (2006)
High NO_3_	10 µmol L^−1^	Snowmelt	Hodson (2006)
3N-BBM	8.8 mmol L^−1^	Culture collection growth media	Bischoff and Bold (1963), Andersen (2005)

Snow algal responses to nitrate availability were assessed under both low (50 µmol photons m^2^ s^−1^) and higher (500 µmol photons m^2^ s^−1^) light intensities. These were achieved using the ‘Warm White’ setting of the multicultivators, with wavelengths peaking at 450 and 600 nm with no UV provision, on a 16:8 h L:D cycle; replicating illumination timescales apparent during the spring ablation seasons in the polar regions where our algal strains were isolated from. Though reduced as compared to expected *in situ* light conditions (∼ 2000 µmol photons m^−2^ s^−1^), the light treatments applied here were selected to allow quantification of responses across an order-of-magnitude difference in light intensity, while maintaining viable cultures in the lab. A total of *N* = 8 replicates were established per algal strain, nitrate concentration and light treatment. Prior to the inoculation of cultures, cells that had been growing in 3N-BBM were rinsed in triplicate with treatment media (Low, Medium, or High NO_3_ concentration) before final inoculation into multicultivators. For this, cultures were centrifuged in an Eppendorf 5804R centrifuge at 300 RCF (Eppendorf, Hamburg, Germany) at 4°C for 7 min, the supernatant removed, and the pelleted algae resuspended in the selected treatment media. This process was repeated three times, before inoculating 2 mL of the rinsed algae into 70 mL media.

Throughout incubations, sampling for determination of algal abundance (cells mL^−1^) and biovolume (µm^3^ per cell) was conducted a minimum of three times per week on 50 µL of each culture sampled using a sterile plastic tip on a manual displacement pipette after thorough homogenization. Homogenization was achieved using 20 mm stirrer bars (VWR International, Lutterworth, UK), sterilized before addition, and a Fisherbrand mini magnetic stirrer (Thermo Fisher Scientific, Massachusetts, USA). Cell abundance and biovolume (see below) were used to identify exponential and stationary growth phases of each strain/nitrate treatment, during which destructive sampling of *N* = 4 replicates was undertaken for photophysiological and stoichiometric analyses (see below).

### Incubation characterization

#### Abundance, biovolume, and growth determination

Throughout both sets of incubations, cell abundance (cells mL^−1^) and biovolume (µm^3^) were monitored a minimum of three times per week on 50 µL subsamples fixed with 1% glutaraldehyde final concentration. Cell abundance was measured by counting cells on a modified Fuchs Rosenthal Haemocytometer (0.2 mm by 1/16 mm^2^; Hawksley, Lancing, UK) using a bright field Olympus BX41 microscope (Germany). Additional to cell counts, images of each sample were taken at 10x and 40x magnification with a MicroPublisher 6 CCD camera attachment (Teledyne Photometrics, USA) and the width and radius of 15 cells measured per replicate using ImageJ software and calculated to biovolume per cell (µm^3^ cell^−1^) assuming each strain to be a prolate spheroid (Hillebrand et al. 1999). To provide an estimate of the total algal biovolume of each culture per time step (µm^3^ per mL^−1^), the mean biovolume per cell was calculated across all 15 cells measured and multiplied by the cellular abundance. To model and summarize the growth of each culture throughout incubations, the ‘SummarizeGrowth’ function of the ‘Growthcurver’ R package v.0.3.1 (Sprouffske and Wagner 2016) was used to fit a logistic regression to either algal abundance (cells mL^−1^) or total biovolume (µm^3^ mL^−1^) datasets (A) in relation to incubation time (h) as:


\begin{eqnarray*}
{{\mathrm{A}}_{\mathrm{t}}} = \frac{{\mathrm{K}}}{{1 + \left( {\frac{{{\mathrm{K}} - {\mathrm{A}}}}{{{{\mathrm{A}}_0}}}} \right){{\mathrm{e}}^{ - {\mathrm{rt}}}}}}.
\end{eqnarray*}

(Equation (1); Sprouffske and Wagner 2016)

The function identifies the optimal values for the maximum possible abundance (carrying capacity; *K* ) given the cellular abundance (cells mL^−1^ ) or total biovolume (µm^3^ mL^−1^ ) measured throughout the incubation. In addition, the specific growth rate (µ) of cultures during their exponential phase of incubations was calculated from logistic regression trajectories above.


\begin{eqnarray*}
{\mathrm{Specific\,\,growth\,\,rate\,\,}}\left( {\mathrm{\mu }} \right) = \frac{{{\mathrm{ln}}\left( {\frac{{{{\mathrm{N}}_2}}}{{{{\mathrm{N}}_1}}}} \right)}}{{{{\mathrm{t}}_2} - {{\mathrm{t}}_1}}}.
\end{eqnarray*}

(Equation (2); Krzemińska et al. 2014)

Where N_1_ and N_2_ are the cell abundance (cells mL^−1^) or total biovolume (µm^3^ mL^−1^) at times 1 and 2, respectively.

#### Photophysiology determination

Rapid light response curves (RLCs; Perkins et al. 2006) were performed using PAM fluorometry to characterize the photophysiological responses of all species/strains during exponential and stationary growth phases of both sets of incubations. Measurements were conducted on 3 mL subsamples of each culture using a Walz Water-PAM fluorometer with attached red-light emitter/detector cuvette system and stirrer (Walz GmBH). Each sample was dark adapted for a minimum of 5 min under incubation temperature prior to RLC measurement. RLCs consisted of nine sequential light steps of 20 s duration ranging in irradiance from 0 to 2000 µmol photons m^−2^ s^−1^. The maximum quantum efficiency (*F_v_/F_m_*) was calculated from minimum (*F_0_*) and maximum (*F_m_*) fluorescence yields measured in the dark-adapted state during the initial RLC step of 20 s darkness (Consalvey et al. 2005). Electron transport through photosystem II (PSII) was calculated across subsequent light steps in relative units (rETR) assuming an equal division of light between PSI and PSII (Consalvey et al. 2005). Analysis of all RLC data (rETR versus PAR) followed Eilers and Peeters (1988) with calculation of the relative maximum electron transport rate (rETRmax), the maximum light utilization coefficient (α), and the light saturation coefficient (Ek). Nonphotochemical quenching (NPQ) was calculated for each light step after Consalvey et al. (2005) and reported as NPQ(Ek), i.e. the level of NPQ apparent at Ek.

#### Stoichiometry determination

The cellular carbon (C) and nitrogen (N) contents of all algal strains were measured during exponential and stationary growth phases of both sets of incubations to determine stoichiometric responses to growth conditions. In all cases, a subsample of known volume, as well as blanks of each media type, were filtered onto preweighed, precombusted (450°C for 5 h) 13 mm diameter GF/A filters (1.6 µm retention; Cytiva Whatman, Maidstone, UK), which were subsequently frozen at −20°C until analysis. Filters were freeze-dried for 24 h to remove all water, reweighed and wrapped in individual 16 mm tin disks prior to elemental analysis using a Vario PYRO cube^®^ (Elementar, Stockport, UK). The detection limits of elemental concentrations were 0.001% for both elements measured, and the coefficient of variation (CV) for C and N according to 12 replicates of an organic analytical standard (NC Soil Standard 338 40025, cert. 341506, C = 2.31%, N = 0.23%; ThermoFisher Scientific, Bremen, Germany) were 5.32% and 2.94%, respectively. The molar content of carbon and nitrogen per cell was derived from the total recorded carbon and nitrogen area as:


\begin{eqnarray*}
mMol\,\,x\,\,cel{l^{ - 1}} = \frac{{A{r_x}\left( {\frac{{x\left[ \% \right]}}{{100}} \times w} \right)}}{{{A_{tot}}}},
\end{eqnarray*}

where Ar_x_ is the relative atomic mass of nutrient x (e.g. carbon), x [%] is the derived percentage of nutrient x present in a processed sample, w is the total weight of the processed sample, and A_tot_ is the total number of cells filtered onto the processed sample (e.g. cells mL^−1^ × mL filtered). This was then used to calculate the molar C:N ratio.

#### Data analysis

The analysis and plotting of data were completed using R v.4.2.1 (R Core Team 2022). Data were first checked for homogeneity of variance and normal distribution. Two- and three-way analysis of variance (ANOVA) tests were used to compare measured parameters for growth, photophysiology, and stoichiometry between treatments, with *post hoc* Tukey HSD analysis applied to all significant ANOVA results.

## Results and discussion

### Temperature incubations

**Figure 1.**
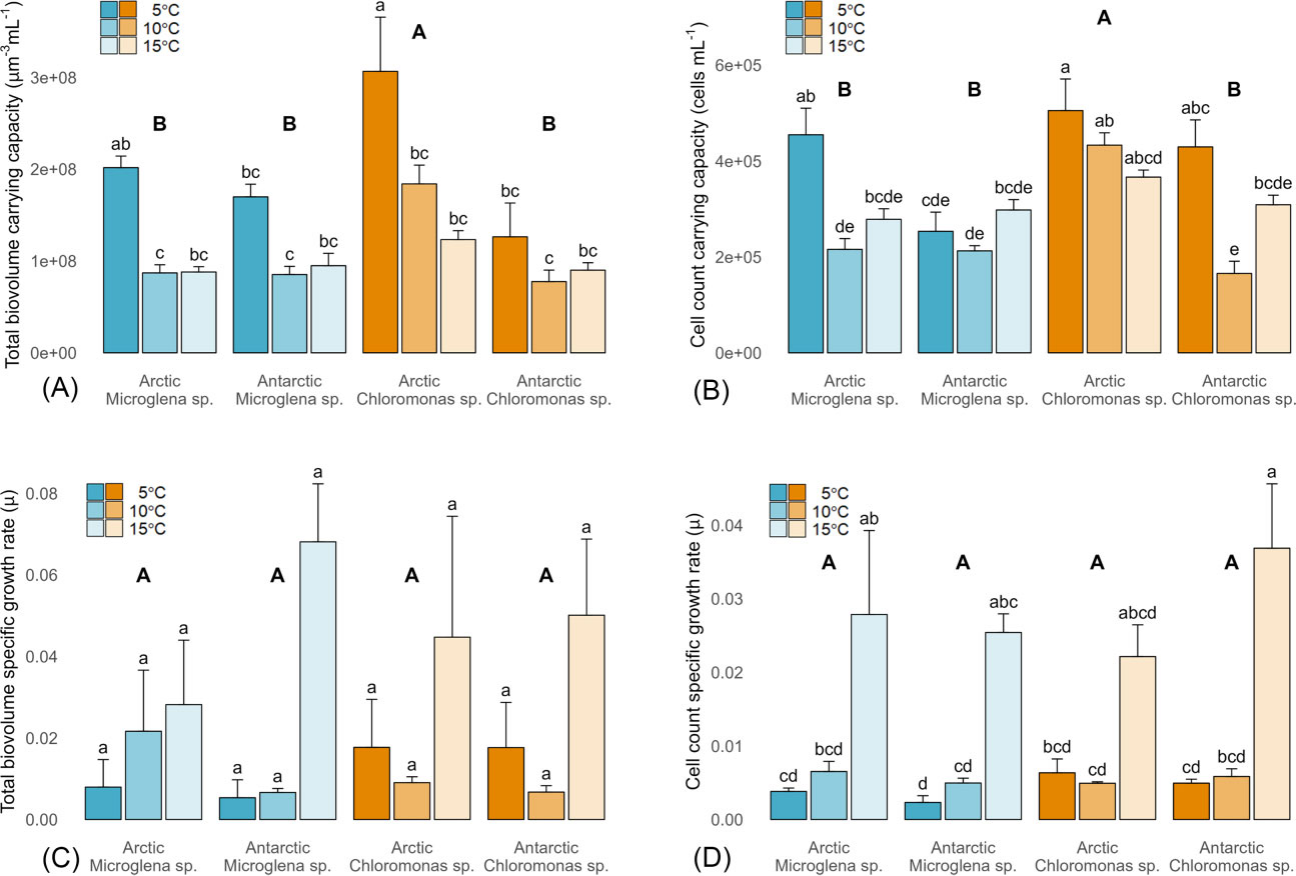
Growth parameters for the temperature incubations. All panels show mean ± SE, *N* = 4. Lower case letters indicate homogenous subsets determined through a two-way ANOVA analysis of respective parameters in relation to temperature and strain. Upper case letters indicate homogenous subsets determined through one-way ANOVA analysis in relation to strain only. **(A)** Biomass carrying capacity — *K* (lc: F6,35 = 1.77, UC: F3,35 = 13.45, *P* < .05). **(B)** Cell count carrying capacity — *K* (lc: F6,36 = 3.87, UC: F3,36 = 13.52, both *P* < .05). **(C)** Biomass specific growth rate during exponential phase — µ (lc: F6,36 = 0.90, UC: F3,36 = 0.16). **(D)** Cell count specific growth rate during exponential phase — µ (lc: F6,36 = 0.69, UC: F3,36 = 0.79).

All snow algal strains demonstrated higher carrying capacities (*K*) but lower specific growth rates (µ) when incubated at 5°C as compared to 10 or 15°C (Fig 1). This trend was most pronounced for total biovolume datasets (µm^3^ mL^−1^), whereby a ∼90% increase was observed in *K* at 5°C as compared to higher temperatures (Fig 1A and B); with exponential phase µ ∼1500% smaller for all strains at 5°C (Fig 1C and D). This indicated a metabolism that maximized total biomass production (*K*) through slower specific growth rates (µ) over longer periods at lower temperatures for all snow algal strains incubated here; consistent with Hoham (1975), who observed 20x higher *K* at 5°C compared to 15°C for *Chloromonas pichinchae* during comparable laboratory incubations. Hoham et al. (2008) also recorded similar comparisons in *K* between 5 and 15°C for *Chloromonas tughillensis*, another *Chloromonas* species with a low temperature optimum.

To be considered a true psychrophile, microbes should possess the ability to grow at 0°C, with optimal growth at or below 15°C (Morita 1975, Finster 2008, Cvetkovska et al. 2017). Psychrotolerant species are generally defined as showing growth between 7 and 35°C, with optimal growth below 20°C (Finster 2008); though strict definitions are still debated (Hoham and Remias 2020, Hüner et al. 2022). For oligotrophic eukaryotic communities, carrying capacity (*K*) has been proposed as the most appropriate measure of optimal growth given that microorganisms are governed by both thermodynamics and biology (Feller and Gerday 2003). Here, increasing temperature increases individual reaction rates, but metabolic processes that are heat labile are compromised (Loppes et al. 1996, Feller and Gerday 2003, Cavicchioli 2016). Accordingly, maximal *K* at 5°C during the present study supports the psychrophilic nature of our incubated snow algal strains, despite the higher specific growth rates (µ) observed at 15°C. In contrast, other studies have defined related snow algal species as psychrotolerant (Seaburg et al. 1981, Lukeš et al. 2014, Barcytė et al. 2018) but these used different metrics to define optimal growth.

**Figure 2.**
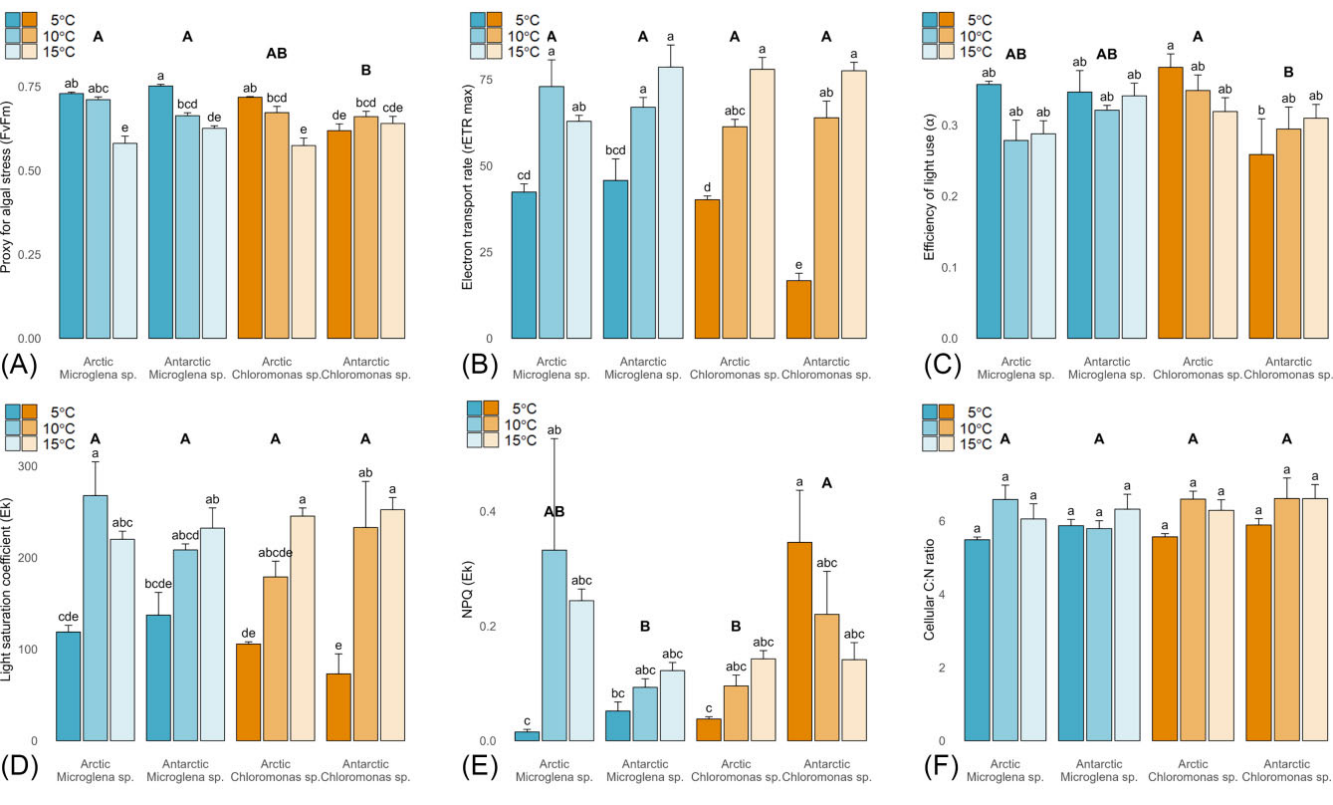
Photophysiology and stoichiometry parameters for the exponential phase of the temperature incubations. All panels show mean ± SE, *N* = 4. Lower case letters indicate homogenous subsets determined through a two-way ANOVA analysis of respective parameters in relation to temperature and strain. Upper case letters indicate homogenous subsets determined through one-way ANOVA analysis in relation to strain only. **(A)** Proxy for algal stress — Fv/Fm (lc: F6,36 = 9.14, UC: F3,36 = 4.56, *P* < .05). **(B)** Electron transport rate — rETR max (lc: F6,34 = 5.89, *P* < .05, UC: F3,34 = 2). **(C)** Efficiency of light use — alpha (lc: F6,34 = 1.69, UC: F3,34 = 3.91, *P* < .05). **(D)** Light saturation coefficient — Ek (lc: F6,34 = 1.93, UC: F3,34 = 3.91, *P* < .05). **(E)** NPQ at Ek (lc: F6,33 = 3.40, UC: F3,33 = 5.15, both *P* < .05). **(F)** Cellular C:N ratio (lc: F6,36 = 0.87, UC: F3,36 = 0.84).

Photophysiology datasets further confirmed the low temperature preference of incubated snow algal strains and indicated the mechanism of reduced µ under lower growth temperatures. Maximum quantum yields in the dark-adapted state (Fv/Fm; an inverse proxy of stress in microalgae; Consalvey et al. 2005) were significantly increased (∼115% ± 7%) at lower incubation temperatures relative to 15°C during the exponential growth phase for all strains except Antarctic *Chloromonas* sp. (Fig 2A). In contrast, maximum electron transport rates (rETRmax) were decreased by ∼50% at 5°C as compared to 10 and 15°C (Fig 2B) for all strains; consistent with patterns previously observed for *C*. cf. *nivalis* (Remias et al. 2005, Lukeš et al. 2014). Taken together, these datasets demonstrated the impact of decreased temperatures on downstream metabolic carbon sinks as compared to the overall capacity for photochemistry of our incubated snow algal strains (Ensminger et al. 2006). Photoautotrophs generally strive to maintain an equilibrium between energy supply (electron transport) and energy utilization (carbon fixation) as environmental forcings change, such that with reduced temperatures, the efficiency of the light-independent reactions of photosynthesis can be suppressed (Maxwell et al. 1994). This results in cold temperatures mimicking the effect of high light on algal photophysiology, inducing a corresponding suppression of electron transport (Morgan-Kiss et al. 2006). Interestingly, three of our four strains showed a greater induction of NPQ at higher temperatures during the present study (excepting Antarctic *Chloromonas* sp.; Fig 2E), likely reflecting the higher levels of electron transport apparent at these temperatures (Serôdio and Lavaud 2011, Blommaert et al. 2021). In contrast, the Antarctic *Chloromonas* sp. showed decreased Fv/Fm, rETRmax and elevated NPQ at lower temperatures, indicating a greater degree of stress for this species at 5°C.

The C:N ratio of all snow algal strains averaged 5.7 ± 0.3 during nutrient replete temperature incubations (Fig 2F), approaching the Redfield C:N stoichiometry of 6.6 (Redfield 1958), with no significant change relative to temperature despite the variable growth rates (µ) and carrying capacities (*K*) apparent across incubations. In the marine environment, numerous studies have demonstrated how marine phytoplankton preferentially grow where the nutrient availability matches their optimal requirements (Arteaga et al. 2014), such that variability in marine POM stoichiometry does not reflect species plasticity, but differences in assemblage composition (Quigg et al. 2003, Daines et al. 2014, Garcia et al. 2018). When we compare the growth and stoichiometric responses of snow algae grown here under nutrient replete conditions (temperature incubations) to those approaching *in situ* nitrate concentrations (see below) our data highlights how similar dynamics would not be expected for microalgal communities in snowpack environments.

### Nitrate and light incubations

**Figure 3.**
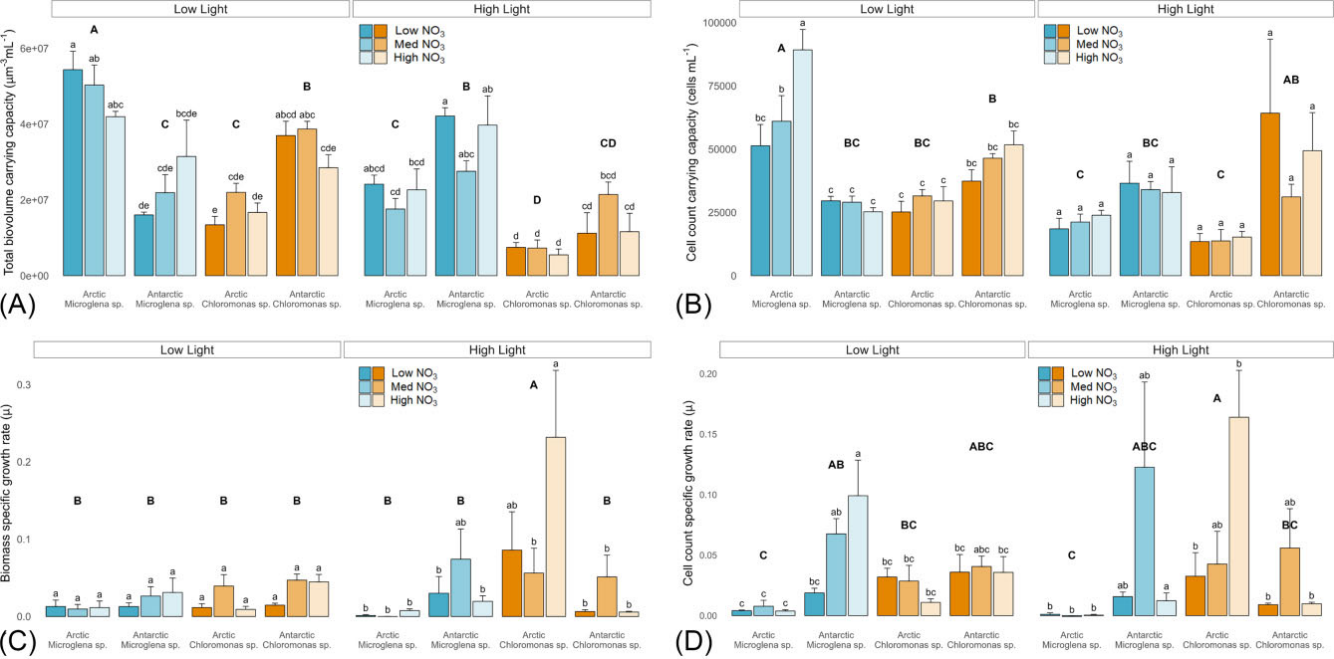
Growth parameters for the nitrate and light incubations. All panels show mean ± SE, *N* = 4. Lower case letters indicate homogenous subsets determined through a two-way ANOVA analysis of respective parameters in relation to strain and nitrate within light treatments. Upper case letters indicate homogenous subsets determined through a three-way ANOVA analysis in relation nitrate, light, and strain. **(A)** Biomass carrying capacity — *K* (lowercase: low light: F6,35 = 2.43; high light: F6,29 = 2.21; both *P* < .05), (uppercase: F3,64 = 27.23, *P* < .05). **(B)** Cell count carrying capacity — *K* (lowercase: low light: F6,36 = 3.17; *P* < .05; high light: F6,32 = 0.52), (uppercase: F3,68 = 11.53, *P* < .05). **(C)** Biomass specific growth rate during exponential phase — µ (lowercase: low light: F6,35 = 1.33; high light: F6,35 = 2.56; *P* < .05), (uppercase: F3,70 = 7.04, *P* < .05). **(D)** Cell count specific growth rate during exponential phase — µ (lowercase: low light: F6,36 = 3.46; high light: F6,35 = 3.62; both *P* < .05), (uppercase: F3,71 = 3.71, *P* < .05).

When grown at 4°C across a range of nitrate concentrations designed to approximate *in situ* nitrate availability (1–10 µmol L^−1^), snow algal growth (both µ and *K*) was significantly decreased as compared to growth under nutrient replete (3N-BBM) conditions, with the decrease in *K* exacerbated by higher-light (500 µmol photons m^−2^ s^−1^) conditions. Under low light (50 µmol photons m^−2^ s^−1^), biovolume-based carrying capacities (*K*; µm^3^ mL^−1^) were 17.4% ± 9.9% of those apparent under 3N-BBM for the same light and temperature conditions (contrast Fig 3A with Fig 1A and B), despite a relative nitrate availability of just 0.01% – 0.11% 3N-BBM (Table 2). Assuming a linear increase in *K* with nitrate availability until maximal *K* is achieved (estimated as *K* measured under 3N-BBM), 100% *K* would be apparent at just 0.64% of 3N-BBM nitrate availability, i.e. 56 µmol L^−1^. This is ~5 – 10x higher than concentrations previously measured in snowpacks (Hodson 2006, Larose et al. 2013), though 10x lower than optimal nitrate requirements of nonsnowpack inhabiting Chlorophytes (Corredor et al. 2021). Under high light, biovolume-based *K* was ∼20% lower than that achieved under low light across incubations (Fig 3A), suggesting that higher light served to further restrict total biomass production under conditions of low nitrate availability.

**Figure 4.**
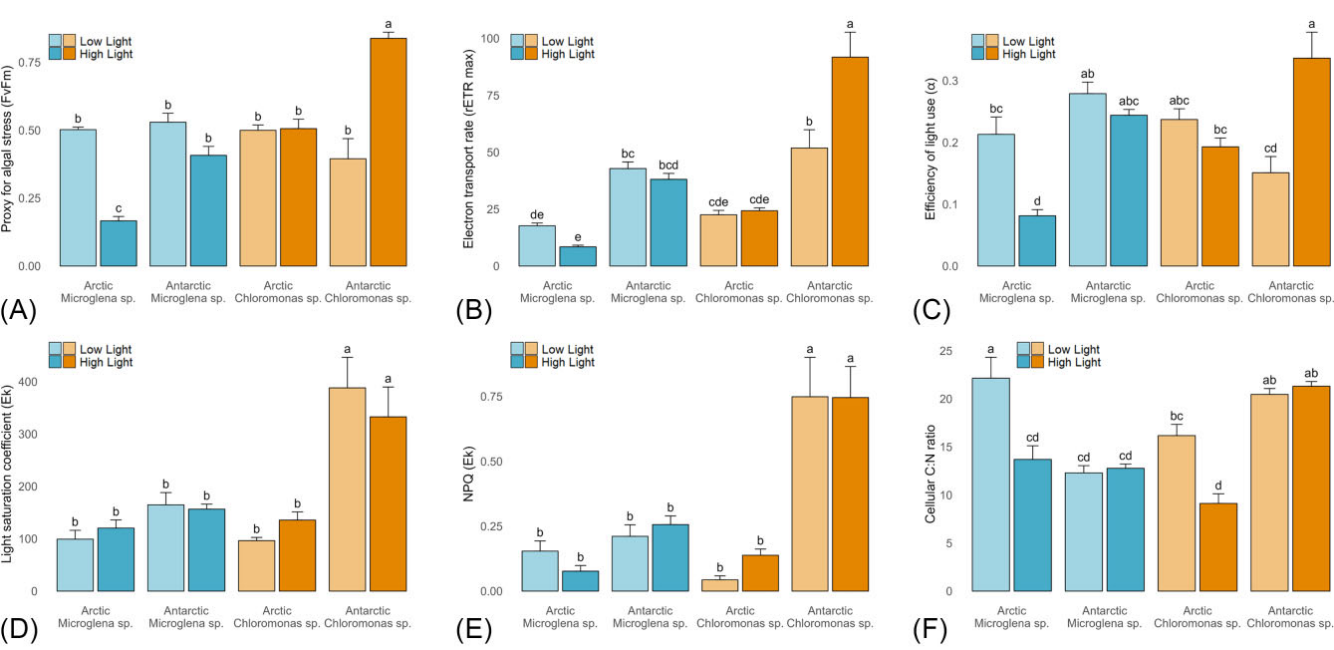
Photophysiology and stoichiometry parameters for the exponential phase of the nitrate and light incubations. All panels show mean ± SE, *N* = 12. Lower case letters indicate homogenous subsets determined through a two-way ANOVA analysis of respective parameters in relation to light intensity and strain. **(A)** Proxy for algal stress — Fv/Fm (F3,87 = 41.51, *P* < .05). **(B)** Electron transport rate — rETR max (F3,86 = 10.09, *P* < .05). **(C)** Efficiency of light use — alpha (F3,87 = 17.16, *P* < .05). **(D)** Light saturation coefficient — Ek (F3,86 = 0.86). **(E)** NPQ at Ek (F3,78 = 0.43). **(F)** Cellular C:N ratio (F3,88 = 9.15, *P* < .05).

Under low light, low nitrate conditions, C:N ratios were ∼200% ± 90% higher for all strains as compared to C:N observed under 3N-BBM at 5°C, averaging 17.8 ± 5.9 (contrast Fig 2F with Fig 4F). Cultured snow algal C:N stoichiometry here was thus consistent with *in situ* values recorded during snow algal blooms under comparable nitrate concentrations (0.5 – 7µmol L^−1^), whereby C:N ranged 16 – 33 (Spijkerman et al. 2012). Taken together, these data intonate that snow algae blooming within snowpacks are likely not growing at their optimum C:N availabilities, but rather showing stoichiometric plasticity relative to ambient nitrate availability. This contrasts with expectations for marine microalgal communities, whereby taxonomic differences in community composition are believed to be responsible for varying POM stoichiometry as opposed to plastic responses of individual species themselves (Quigg et al. 2003, Arteaga et al. 2014, Daines et al. 2014, Garcia et al. 2018). Despite the clear growth preferences for lower temperatures shown here, C:N responses indicated that snow algal species may not be adapted to the oligotrophic nature of their snowpack environments in relation to nitrate availability. Recently, Williamson et al., (2021) demonstrated low N and P cellular quotients of Streptophyte glacier algae sampled from surface ice of the Greenland Ice Sheet, concluding that lower N and P cellular requirements likely reflected adaptation of glacier to their oligotrophic icy environment. However, given that glacier algae have only recently been brought into culture (Remias and Procházková 2023), Williamson et al. (2021) were not able to confirm this by culturing glacier algae under nutrient replete conditions and observing cellular responses. Our data show clear stoichiometric plasticity of multiple snow algal strains when grown under snowpack nitrate concentrations as opposed to strains adapted to overall lower nutrient concentrations. Though it should be noted that all strains were capable of growth at these nitrate concentrations, with no differences apparent in photophysiology or stoichiometric responses across the 1 – 10µmol L^−1^ range of nitrate treatments employed.

A total of three of the four strains (excluding Arctic *Microglena* sp.) demonstrated higher specific growth rates (µ) under the higher light treatment (500 µmol photons m^−2^ s^−1^); though *K* remained depressed at higher-light. For these three strains, abundance-based specific growth rates (µ) were higher but more variable (0.053 ± 0.077) than under low light (0.032 ± 0.034) (Fig 3C). This is supported by both generally consistent or increased Fv/Fm and rETRmax (Fig 4A and B) across these three strains between the low (Fv/Fm: 0.50 ± 0.14; rETRmax: 39.04 ± 21.07) and high-light conditions (Fv/Fm: 0.58 ± 0.21; rETRmax: 50.21 ± 35.65). These data suggest that any photoinhibition induced by the higher light level was not sufficient to exceed photoprotective capabilities of these strains (Mojzeš et al. 2020, Maltsev et al. 2021), allowing for a reduction in stress (Fv/Fm) and higher specific growth rates, indicated by rETRmax and µ.

Intra-specific differences were observed in photophysiological responses between low and high-light conditions. For example, the rETRmax of the Arctic *Microglena* sp. was overall ∼50% that of the Antarctic strain across all light treatments (20.2 ± 6.0 and 47.3 ± 21.0, respectively; Fig 4B). The Arctic *Microglena* sp. also showed a lower efficiency of light use (*α*) under high-light, suggesting photoacclimation through reduction of antenna size to protect PSII from photodamage (Melis 1991), with a comparable rETRmax achieved across the two light treatments for this Arctic strain,. For *Chloromonas* sp. there were also intra-specific differences. The Arctic strain maintained comparable Fv/Fm between the two light intensities, while the Antarctic strain's Fv/Fm increased by ∼90%–150% under high light (Fig. 4A). This suggested that our Antarctic *Chloromonas* sp. preferentially grew under higher light, which was not apparent for either *Microglena* sp.. NPQ did not vary between light treatments for Antarctic *Chloromonas* sp., but was ∼600% ± 200% higher overall as compared to the other three strains (Fig 4E), suggesting a greater dependence on NPQ processes in this strain. The capacity of another closely related snow alga, *Chloromonas kaweckae*, to cope with higher light conditions has also been noted *in situ*, where it has been found in the upper 3 cm of the snowpack (Procházková et al. 2023).

This study has highlighted diversity in studied snow algal strain responses to environmental stressors (temperature, light, and nitrate availability), and how they behave differently to other microalgal communities. Nutrient replete temperature incubations demonstrated the psychrophilic nature of these strains, with carrying capacity (*K*) taken as an appropriate metric of growth. Incubations also demonstrated how colder temperatures mimic the effects of high light on algal photophysiology. Under nutrient replete conditions, the C:N ratio of studied strains approached Redfield stoichiometry (6.6), whereas significantly elevated C:N (17.8 ± 5.9) was evident when strains were grown in nitrate concentrations that reflected snowpack conditions. These data highlight the plasticity in snow algal cellular stoichiometry and suggest communities growing *in situ* are not receiving their optimal nutrient requirements. Preliminary estimates indicate optimal nitrate concentrations 5 – 10x that found in snowpacks, but 10x lower than that of other nonsnowpack inhabiting chlorophytes. Higher-light conditions under *in situ* nitrate concentrations elucidated intra-specific differences in algal photophysiology, with suppressed parameters for the Arctic *Microglena* sp. and preferential responses from the Antarctic *Chloromonas* sp. strain.

## Supplementary Material

fiad088_Supplemental_Figure
