# Supplementary figures and images for: Adaptation versus plastic responses to temperature, light, and nitrate availability in cultured snow algal strains
Source: FEMS Microbiol Ecol. 2023 Aug 8;99(9):fiad088. doi: 10.1093/femsec/fiad088 (PMC10481995; doi:10.1093/femsec/fiad088)

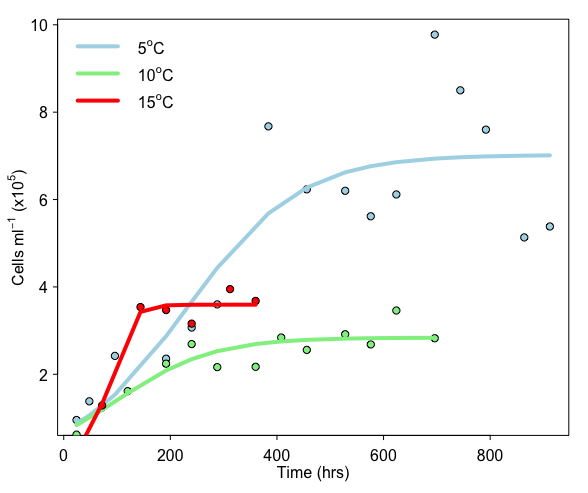

Supplement: fiad088_Supplemental_Figure [file fiad088_supplemental_figure.zip › Supp_data example_growth_curves.tiff]
